# Rotatory Movements of the Yolk in the Ovum of Mammalia, during Its Passage through the Fallopian Tube

**Published:** 1842-03

**Authors:** 


*Rotatory Movements of the Yolk in the Ovum of Mammalia, during its passage through the Fallopian tube.*—The attention of Prof. Bischoff of Heidelberg was called to this phenomenon by an observation of Dr. Martin Barry in the Philosopical Transactions for 1839. Dr. Barry there mentions having on one occasion met with an elliptical vesicle (filled with a transparent fluid in which were small elliptical granules), adherent to the mucous membrane of the Fallopian tube. In the centre of this vesicle was a mulberry-like body which continued rotating itself for half an hour, the rotation subsiding by degrees into a tremulous motion. Professor Bischoff was of opinion that this vesicle was an ovum and the rotating body its yolk, and he adduces an observation in corroboration of this suppposition.

Having carefully laid open, with a pair of scissors, the Fallopian tubes of a rabbit, which had been placed with a male during the previous eight days, he found four ova close together about the middle of the tube of the left side. Within the zona pellucida of these ova was the yolk which, however, did not completely occupy its area, but between the yolk and the inner surface of the zona a transparent fluid intervened. In this fluid each yolk ball continually rotated on its own axis, in the direction from the uterus to the ovary. On examining the ova with a power of 800 diameters, the surface of the yolk was seen to be furnished with very minute ciliæ, by the vibration of which this movement was effected. Professor Bischoff convinced himself, by very careful examination, that the ova themselves remained perfectly still during the continuance of this rotatory motion of the yolk, which ceased on the preparation being moistened, in order to prevent the ova from drying up.

In this phenomenon Professor Bischoff sees a fresh point of coincidence between the processes of developement in mammalia and in other animals, since similar rotatory movements of the yolk have been observed in the ova of mollusca and polyps, and recently by Von Siebold and Ehrenberg in those of medusa aurita.—*L. and E. Monthly Jour. Med. Sci.*, Oct. 1841, from *Muller's Archives*, Heft. 1, 1841.

